# Metabolic effects of a 13-weeks lifestyle intervention in older adults: The Growing Old Together Study

**DOI:** 10.18632/aging.100877

**Published:** 2016-01-25

**Authors:** Ondine van de Rest, Bianca A.M. Schutte, Joris Deelen, Stephanie A.M. Stassen, Erik B. van den Akker, Diana van Heemst, Petra Dibbets-Schneider, Regina. A. van Dipten-van der Veen, Milou Kelderman, Thomas Hankemeier, Simon P. Mooijaart, Jeroen van der Grond, Jeanine J. Houwing-Duistermaat, Marian Beekman, Edith J.M. Feskens, P. Eline Slagboom

**Affiliations:** ^1^ Division of Human Nutrition, Wageningen University, 6700 EV Wageningen, The Netherlands; ^2^ Department of Molecular Epidemiology, Leiden University Medical Center, 2300 RC Leiden, The Netherlands; ^3^ Department of Gerontology and Geriatrics, Leiden University Medical Center, 2300 RC Leiden, The Netherlands; ^4^ The Delft Bioinformatics Lab, Delft University of Technology, 2628 CD Delft, The Netherlands; ^5^ Department of Radiology, Leiden University Medical Center, 2300 RC Leiden, The Netherlands; ^6^ Division of Analytical Biosciences, Leiden Academic Centre for Drug Research, Leiden University, Leiden 2300 RA, The Netherlands; ^7^ Department of Medical Statistics, Leiden University Medical Center, 2300 RC Leiden, The Netherlands

**Keywords:** lifestyle intervention, older adults, caloric restriction, physical activity, metabolic health, healthy ageing

## Abstract

For people in their 40s and 50s, lifestyle programs have been shown to improve metabolic health. For older adults, however, it is not clear whether these programs are equally healthy. In the Growing Old Together study, we applied a 13-weeks lifestyle program, with a target of 12.5% caloric restriction and 12.5% increase in energy expenditure through an increase in physical activity, in 164 older adults (mean age=63.2 years; BMI=23-35 kg/m^2^). Mean weight loss was 4.2% (SE=2.8%) of baseline weight, which is comparable to a previous study in younger adults. Fasting insulin levels, however, showed a much smaller decrease (0.30 mU/L (SE=3.21)) and a more heterogeneous response (range=2.0-29.6 mU/L). Many other parameters of metabolic health, such as blood pressure, and thyroid, glucose and lipid metabolism improved significantly. Many ^1^H-NMR metabolites changed in a direction previously associated with a low risk of type 2 diabetes and cardiovascular disease and partially independently of weight loss. In conclusion, 25% reduction in energy balance for 13 weeks induced a metabolic health benefit in older adults, monitored by traditional and novel metabolic markers.

## INTRODUCTION

Worldwide, the proportion of older and highly aged people in the population is rising fast [1]. Metabolic and physical health generally decline among older adults, be it in a highly heterogeneous fashion [2]. Hence, there is an urge to stimulate healthy ageing among the increasing group of older adults. Metabolic health can successfully be improved by lifestyle changes, such as dietary restriction and/or increased physical activity [3-9], thereby reducing the risk for cardiovascular disease (CVD). An example of a lifestyle intervention showing this metabolic improvement in young adults (28-45 years of age) is the CALERIE study, a 6-month lifestyle intervention reducing energy balance by 25% in 12 overweight individuals (body mass index (BMI) 25-30 kg/m^2^) [6]. As yet, it is unclear whether a 25% reduction in energy balance likewise improves metabolic health in older adults and is feasible in this age group.

Poor metabolic health is generally marked by high levels of total cholesterol, glucose, insulin, triglycerides, and blood pressure and low levels of HDL cholesterol, free triiodothyronine (fT3), and adiponectin [10-13], except in highly aged individuals (above 75 years) [14,15]. Remarkably, the majority of parameters of poor metabolic health inversely associate with familial longevity, as shown by comparison of middle-aged offspring of long-lived subjects and their spouses [16-19]. Besides clinical markers, metabolic health can also be monitored by novel technologies, such as Nuclear Magnetic Resonance (NMR), which are able to measure large numbers of metabolites in an affordable and standardized way. Distinct profiles of metabolites have been demonstrated to associate with intake of specific food components [20,21], (future) type 2 diabetes (T2D) [22-24] and CVD [18,25,26], showing the potential of metabolomics to monitor metabolic health. However, it has not yet been established which optimal set of markers monitors the metabolic effects of a lifestyle change in older adults.

In the Growing Old TOgether (GOTO) study we investigated the effect of a lifestyle intervention in older adults by both clinical and metabolomic profiles. Participants reduced energy balance by 25% for 13 weeks, targeted by 12.5% reduction in caloric intake and 12.5% increase in physical activity, corresponding to one of the three intervention conditions previously applied in the CALERIE study [6]. In CALERIE, 12 participants received this intervention. The GOTO study consisted of 164 individuals (mean age 63.2 years) with a BMI of 23-35 kg/m^2^, which are mostly couples of whom one was member of a longevity family and the other their spouse. Since fasting insulin was one of the markers that showed a reduction within three months in the CALERIE study [6], this parameter was used as our primary outcome. In addition, we measured the response to the intervention by other established markers of metabolic health, state-of-the-art metabolic profiles measured with Hydrogen-1 NMR (^1^H-NMR), and quality of life (QoL).

## RESULTS

### Longevity family members and controls are largely similar on baseline

**Figure 1:**
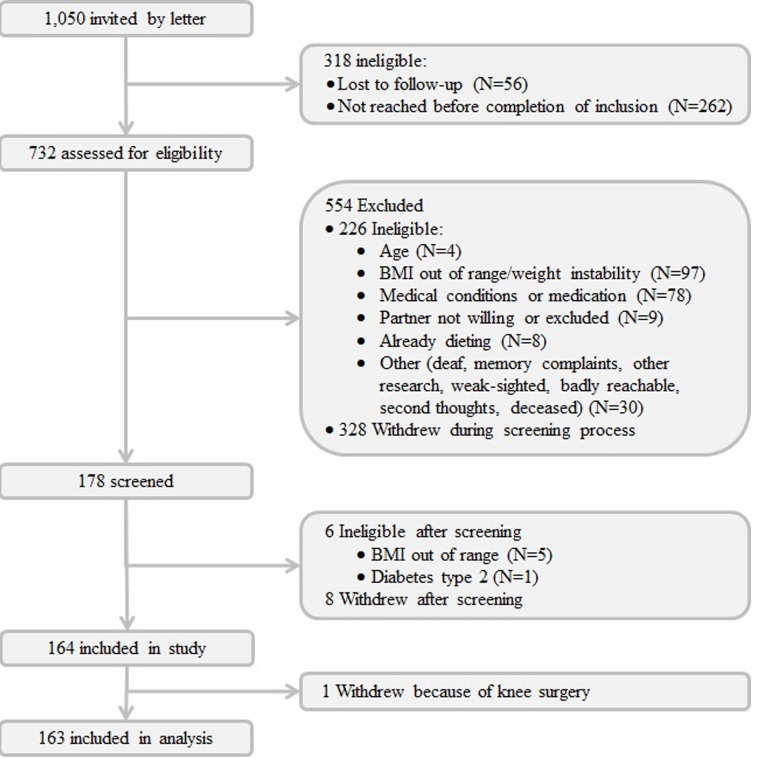
Flow chart of participants in the trial

Of the 164 individuals who started the intervention study, one dropped out prior to completion of the study (Fig. 1). A selection of the clinical baseline characteristics of the participants according to familial background, i.e. longevity family member or control, is depicted in Table 1 (complete clinical baseline characteristics are provided in Supplementary Table 1A). Baseline characteristics of ^1^H-NMR metabolites are shown in Supplementary Table 1B. Gender differences were observed for many parameters. In contrast to the Leiden Longevity Study as a whole, in which many metabolic parameters differ significantly between longevity family members and controls [16,18,19], we observed only few significant differences in the parameters at baseline between the small groups included in the GOTO study. Therefore, we studied the effects of the intervention in both groups combined.

**Table 1:** Baseline characteristics of parameters of body composition, health and functioning, and diagnostic measurements

Characteristic		n	Longevity family members	n	Controls
Women, n (%)			39 (43.3)		42 (56.8)
Age, mean (SD) [range], years		90	63.4 (5.4) [49.1-75.1]	74	62.4 (6.1) [46.7-73.5]
**Body composition, mean (SD) [range]**					
Weight, kg		89	79.8 (9.6) [62.5-105.7]	73	79.0 (10.2) [60.5-102.4]
	Men	50	84.3 (8.0) [67.2-105.7]	31	85.4 (8.1) [70.1-102.4]
	Women	39	74.1 (8.4) [62.5-95.4]	42	74.1 (8.9) [60.5-100.4]
BMI, kg/m^2^		89	27.0 (2.6) [22.9-34.2]	73	26.9 (2.4) [22.9-33.5]
Waist circumference, cm		90	96.2 (7.9) [74-122]	74	96.1 (8.2) [77-112]
	Men	51	98.1 (7.4) [80-122]	32	100.1 (6.4) [89-112]
	Women	39	93.6 (7.9) [74-112]	42	93.0 (8.1) [77-111]
**Health and functioning, mean (SD) [range]**					
Systolic blood pressure, mm Hg^a^		65	135.4 (15.9) [111-196]	48	137.8 (17.1) [101-173]
Diastolic blood pressure, mm Hg^a^		65	83.5 (7.4) [64-101]	48	84.7 (9.2) [65-108]
**Medication use, *n* (%)**					
Lipid-lowering agent		90	11 (12.2)	74	18 (24.3)
Antihypertensive agent		90	23 (25.6)	74	26 (35.1)
**Diagnostic measurements, mean (SD) [range]**					
Fasting glucose, mmol/L		90	5.0 (0.5) [3.6-6.5]	74	5.0 (0.6) [4.0-7.6]
Fasting insulin, mU/L^b^		90	9.4 (5.1) [2.0-29.6]	74	9.0 (3.9) [2.0-22.6]
HOMA-IR		88	1.2 (0.6) [0.4-3.8]	72	1.2 (0.5) [0.4-2.7]
Total cholesterol, mmol/L^c^		79	5.5 (1.0) [3.3-8.6]	56	5.5 (1.0) [3.2-8.0]
HDL cholesterol, mmol/L^c^		79	1.6 (0.4) [0.6-3.1]	56	1.4 (0.4) [0.6-2.3]
	Men	43	1.4 (0.3) [1.0-2.0]	23	1.1 (0.2) [0.6-1.6]
	Women	36	1.7 (0.5) [0.6-3.1]	33	1.6 (0.3) [1.2-2.3]
LDL cholesterol, mmol/L^c^		79	3.5 (0.8) [1.8-6.4]	56	3.4 (0.9) [1.6-6.0]

a Individuals using antihypertensive agents were removed before analysis.

b Natural log transformed parameter was used for analysis.

c Individuals using lipid-lowering agents were removed before analysis.

Parameters were analysed separately in men and women if there was a significant gender-difference at baseline. BMI, body mass index; HOMA-IR, homeostatic model assessment - insulin resistance; HDL, high density lipoprotein; LDL, low density lipoprotein.

### Intervention improves body composition and metabolic health

**Figure 2:**
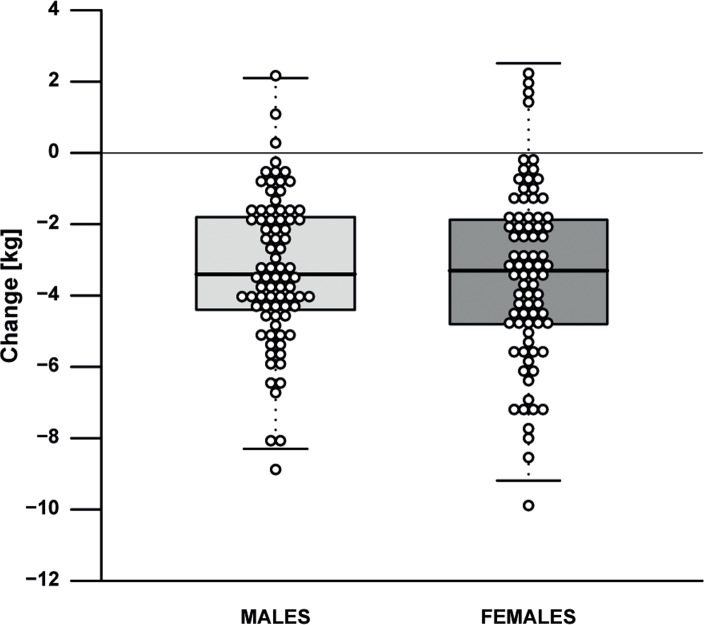
Effect of the intervention on weight by gender

**Figure 3:**
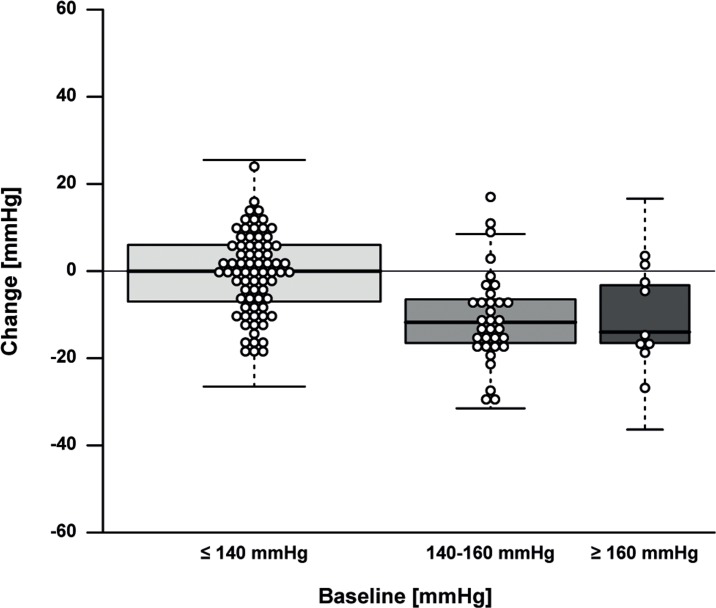
Effect of the intervention on systolic blood pressure by baseline systolic blood pressure

The effect of the 13-weeks lifestyle change on clinical parameters is depicted in Table 2 and Supplementary Table 2A. For the primary outcome, i.e. fasting insulin, a minor mean (SE) decrease of 0.30 mU/L (3.21 mU/L) and a considerable heterogeneity (range −11.5-10.5 mU/L) was observed (Supplementary Fig. 1). Measures of body composition generally improved, as shown by a mean weight loss of 3.3 kg (0.18 kg), i.e. 4.2% (2.8%) of baseline weight (Fig. 2), a body fat mass decrease of 11.7% (8.9%), and a fat free mass decrease of 0.7 kg (0.1 kg). Measures of health and functioning showed a significant decrease of 4.3 mmHg (1.0 mmHg) in systolic and 1.7 mmHg (0.6 mmHg) in diastolic blood pressure. We noted that the changes in weight and systolic blood pressure (Fig. 3), but not in insulin levels, were dependent on baseline levels.

**Table 2:** Effects of the intervention on parameters of body composition, health and functioning, and diagnostic measurements

Characteristic, mean (SE)		n	Difference	P-value^a^
**Body composition**				
Weight, kg		161	−3.34 (0.18)	<0.001
	Men	80	−3.42 (0.27)	<0.001
	Women	81	−3.25 (0.23)	<0.001
BMI, kg/m^2^		161	−1.13 (0.06)	<0.001
Waist circumference, cm		163	−4.3 (0.4)	<0.001
	Men	82	−4.4 (0.6)	<0.001
	Women	81	−4.2 (0.6)	<0.001
Body fat, %		161	−2.26 (0.16)	<0.001
	Men	80	−2.22 (0.23)	<0.001
	Women	81	−2.29 (0.21)	<0.001
Fat free mass, kg^2^		161	−0.67 (0.10)	<0.001
	Men	80	−0.83 (0.16)	<0.001
	Women	81	−0.51 (0.13)	<0.001
**Health and functioning**				
Systolic blood pressure, mm Hg^b^		113	−4.33 (0.98)	<0.001*
Diastolic blood pressure, mm Hg^b^		113	−1.66 (0.61)	0.007
REE, kcal/day		126	−49.2 (8.0)	<0.001*
	Men	65	−46.59 (11.76)	<0.001
	Women	61	−51.94 (10.79)	<0.001*
Handgrip strength, kg		153	0.38 (0.32)	0.25
	Men	76	0.24 (0.53)	0.65
	Women	77	0.51 (0.38)	0.18
Physical functioning		159	0.14 (0.05)	0.008*
Physical quality of life		157	−0.18 (0.61)	0.77
	Men	82	−0.72 (0.83)	0.39
	Women	75	0.42 (0.92)	0.65
Mental quality of life		157	0.9 (0.70)	0.19
	Men	82	−1.13 (0.84)	0.18
	Women	75	3.13 (1.12)	0.005*
FRS, %		163	−0.51 (0.23)	0.03
	Men	82	−0.65 (0.43)	0.13
	Women	81	−0.37 (0.15)	0.01
**Diagnostic measurements**				
Fasting glucose, mmol/L		163	−0.06 (0.04)	0.16
Fasting insulin, mU/L^c^		163	−0.05 (0.03)	0.04
HOMA-IR		153	−0.03 (0.03)	0.33
Total cholesterol, mmol/L^d^		135	−0.29 (0.06)	<0.001^#^
HDL cholesterol, mmol/L^d^		135	−0.01 (0.02)	0.49
	Men	66	0.04 (0.02)	0.11
	Women	69	−0.06 (0.03)	0.02*
LDL cholesterol, mmol/L^d^		135	−0.26 (0.05)	<0.001^#^
Triglycerides, mmol/L^c,d^		135	−0.04 (0.03)	0.11
fT3, pmol/L		163	−0.14 (0.03)	<0.001^#^
fT4, pmol/L		163	−0.07 (0.09)	0.44
TSH, mU/L^d^		163	−0.04 (0.03)	0.17
DHEAS, nmol/L^c^		163	−0.02 (0.01)	0.20
	Men	82	−0.01 (0.02)	0.47
	Women	81	−0.02 (0.02)	0.28
Leptin, μg/L^c^		163	−0.26 (0.03)	<0.001*
	Men	82	−0.29 (0.04)	<0.001*
	Women	81	−0.23 (0.03)	<0.001*
Adiponectin, mg/L^b^		163	0.04 (0.01)	0.005
	Men	82	0.09 (0.02)	<0.001
	Women	81	−0.01 (0.02)	0.76
IGF-1, nmol/L		163	0.10 (0.24)	0.67
	Men	82	0.36 (0.31)	0.24
	Women	81	−0.17 (0.35)	0.64
IGFBP-3, mg/L		163	−0.05 (0.05)	0.37
IGF-1:IGFBP-3		163	0.004 (0.003)	0.21
	Men	82	0.009 (0.006)	0.14
	Women	81	−0.001 (0.003)	0.82
CRP (high-sensitivity), mg/L^c^		163	−0.11 (0.07)	0.09

* P-value < 0.05 after adjustment for weight loss.

# P-value < 0.001 after adjustment for weight loss.

a P-value refers to difference between baseline and end.

b Individuals using antihypertensive agents were removed before analysis.

c Natural log transformed parameter was used for analysis.

d Individuals using lipid-lowering agents were removed before analysis.

Parameters were analysed separately in men and women if there was a significant gender-difference at baseline. BMI, body mass index; REE, resting energy expenditure; FRS, Framingham risk score; HOMA-IR, homeostatic model assessment - insulin resistance; HDL, high density lipoprotein; LDL, low density lipoprotein; fT3, free triiodothyronine; fT4, free thyroxine; TSH, thyroid stimulating hormone; DHEAS, dehydroepiandrosterone-sulfate; IGF-1, insulin-like growth factor 1; IGFBP-3, insulin-like growth factor binding protein 3; CRP, C-reactive protein.

Resting energy expenditure (REE) significantly decreased with 49.2 kcal/day (8.0 kcal/day). Hand grip strength was not changed by the lifestyle change, but physical functioning, mental QoL in women, and the Framingham Risk Score improved. The diagnostic measures showed a significant decrease in total and LDL cholesterol, HDL cholesterol in women, fT3, and leptin levels and an increase in adiponectin levels in men.

### Plasma metabolite profile changes due to 3 months lifestyle intervention

**Figure 4:**
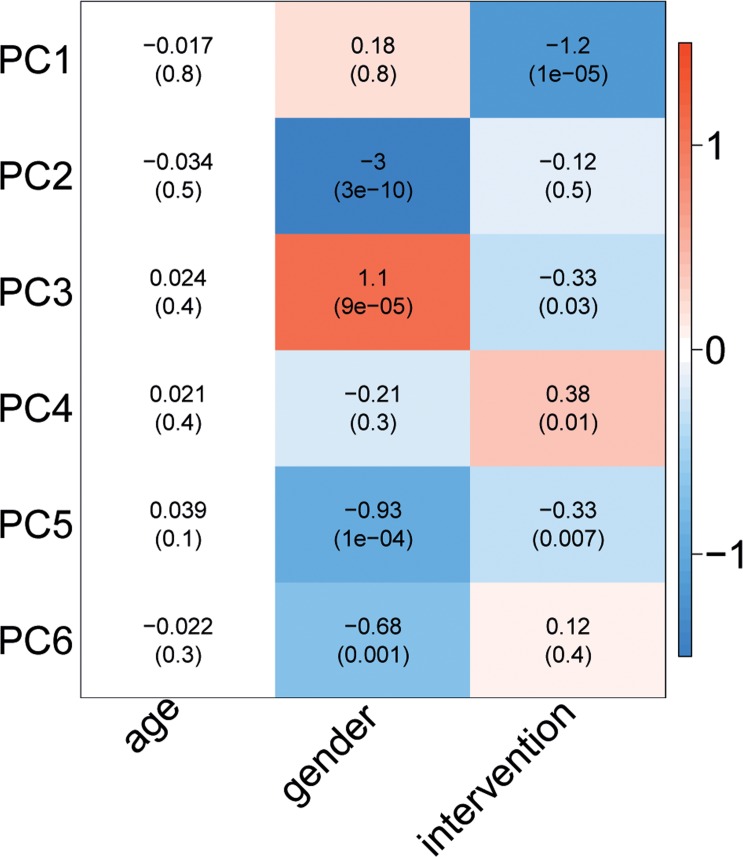
Effect of age, gender, and intervention on ^1^H-NMR metabolite-based PC's. The colour of the blocks represents the magnitude of the effect, while the P-value is mentioned between brackets.

**Figure 5:**
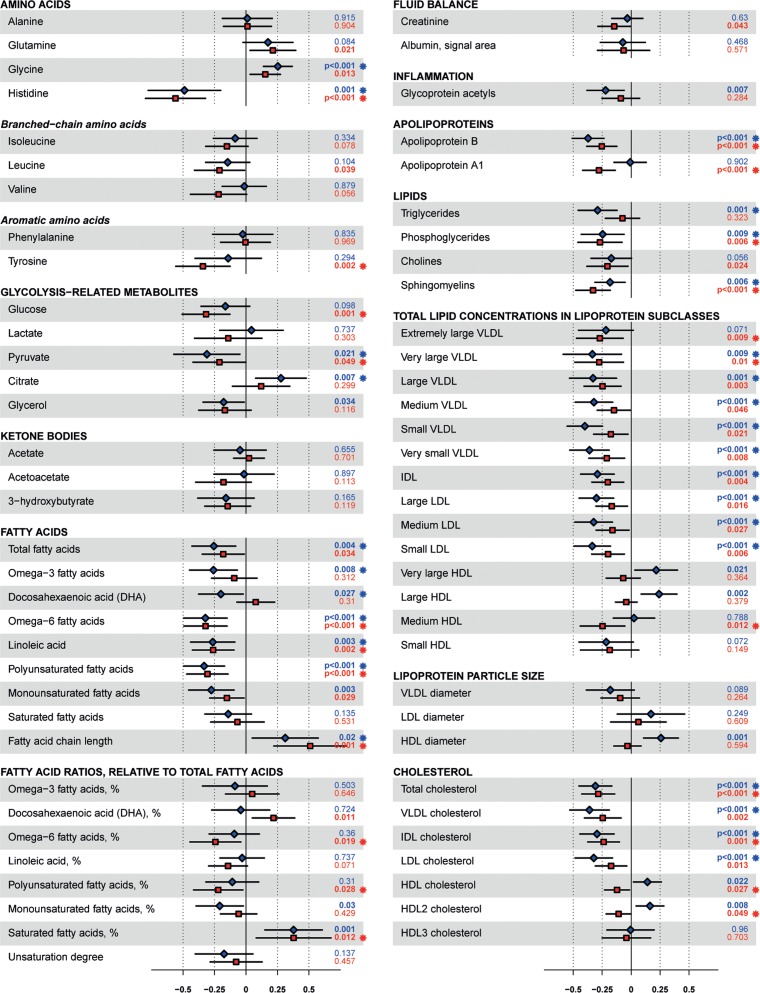
Effects of the intervention on ^1^H-NMR metabolites. Effect sizes are per 1-SD log-transformed metabolite concentration and adjusted for age and gender. Squares indicate mean and error bars denote 95% confidence intervals. Blue squares indicate males, red squares indicate females. Individuals using lipid-lowering agents were removed before analysing fatty acids, fatty acids ratios, apolipoproteins, lipids, total lipid concentrations in lipoprotein subclasses, lipoprotein particle size and cholesterol. VLDL, very low density lipoprotein; IDL, intermediate density lipoprotein; LDL, low density lipoprotein; HDL, high density lipoprotein.

To determine the overall effect of the intervention on the ^1^H-NMR metabolites, we performed Principle Component Analysis. This analysis indicated that a major part of the variation in the metabolites (PC1, explaining 32.4% of the total variance) could be attributed to the effects of the lifestyle intervention (Fig. 4). As shown in Supplementary Fig. 2, the intervention effect, as represented by PC1, coincides with many of the measured ^1^H-NMR metabolites. The effects of the intervention on the single ^1^H-NMR metabolites in fasting blood are depicted in Fig. 5 and Supplementary Table 2B. Multiple amino acids levels changed significantly and include a decrease of the branched-chain amino acid leucine and the aromatic amino acid tyrosine. In addition, the levels of multiple glycolysis-related metabolites, ketone bodies, fatty acids, metabolites involved in fluid balance and inflammation, apolipoproteins, lipid concentrations, and lipoprotein particle sizes showed a significant decrease. Citrate levels, large HDL cholesterol concentrations, and HDL particle size, on the other hand, increased after the lifestyle change. For several HDL-related metabolites we observed an opposite effect of the intervention in men and women.

### Effects of lifestyle intervention at old age (partly) independent of weight loss

To investigate whether the observed response mainly coincides with the change in weight, we adjusted for weight loss. For most of the parameters of health and functioning, diagnostic measurements, and ^1^H-NMR metabolites, adjustment for weight loss reduced the effects of the intervention. However, the changes in fT3, total, VLDL, LDL, and IDL cholesterol, as well as those in phosphoglycerides, cholines, sphingomyelines, and some glycolysis intermediates, remained largely unchanged after this adjustment (Table 2, Supplementary Table 2A and Supplementary Table 2B).

Finally, the beneficial effects on physical functioning and mental QoL in women were also largely independent of weight loss (Table 2 and Supplementary Table 2A). Hence, the effect of the lifestyle change on many of the metabolic parameters and well-being in this study occurs partly or fully independent of the observed loss in weight.

## DISCUSSION

A 13-weeks lifestyle change among older adults aimed at combining 12.5 % decreased energy intake and 12.5% increased physical activity improved parameters of body composition, relevant clinical markers, such as fasting insulin, our primary endpoint, blood pressure, glucose, lipid and thyroid metabolism, and ^1^H-NMR metabolites. In addition, physical functioning and mental QoL in women improved. For most of the parameters, the improvements were, at least partly, independent of weight loss, indicating that we monitored aspects of metabolic health additional to weight loss.

The GOTO study shows that one of the intervention conditions previously pioneered by the CALERIE study in younger and more overweight subjects [6] seems generally feasible, since only one drop-out was observed and metabolic health was generally improved. The older adults in GOTO generally lost weight during the intervention and the mean change in weight was comparable to CALERIE. The mean change in fasting insulin, however, was much smaller in GOTO (0.30 mU/L) than in CALERIE (2.06 mU/L) and the heterogeneity in response was much larger (range −11.5 – 10.5 mU/L and −8 – 2 mU/L, respectively). There could be several explanations for this difference. First, the sample size of the GOTO study is almost 14 times as large as the ‘calorie restriction with exercise’ study group in CALERIE and may have estimated the effect of the intervention more accurately. Second, the baseline and response variation in insulin and other metabolic variables was larger in GOTO than in CALERIE, which might be caused by the higher mean age of our population (mean age 63 years (GOTO) versus 39 years (CALERIE)) and broader range in baseline BMI. Third, because the intervention in GOTO was less controlled, the outcome was likely more heterogeneous. Heterogeneous responses to lifestyle interventions should be further explored and carefully monitored in even larger studies of older adults.

The potential improvement of metabolic health is reflected in the change of clinical parameters as well as metabolites. The observed decrease in leucine, tyrosine, glucose, pyruvate, glycerol, total fatty acids, monounsaturated fatty acids, α-acid glycoprotein, lipid concentrations, and VLDL particle size and increase in fatty acid chain length and citrate imply a decreased future CVD risk based on a previous prospective study, including older adults, using the same ^1^H-NMR assay [26]. This would correspond with reduced FRS in women after the lifestyle change. In addition, the observed decrease in leucine, tyrosine, glucose, 3-hydroxybutyrate, and creatinine and increase in glycine is considered beneficial with respect to (risk of) T2D [22-24]. The observed gender difference in HDL metabolites in response to the intervention may be due to the fact that women already displayed more beneficial levels at baseline as compared to men, while the observed decrease in HDL metabolites in men may be caused by a decrease in alcohol intake [27].

Unlike insulin, blood pressure levels, and the Framingham Risk Score, parameters indicative of lipid and thyroid metabolism, as well as several ^1^H-NMR metabolites, changed largely independent of the reduction in body weight. Such changes may point at the occurrence of metabolic shifts in response to dietary changes and increase in physical activity, exemplified by decreased fT3 levels, which were also observed in the CALERIE study after 3 months [6]. Basically, our data suggest that the effects of the intervention on metabolic health can be monitored by a combination of weight, fT3 levels, and a single ^1^H-NMR metabolite assay.

Next to metabolic health, we observed an improvement of mental health status in women, which was independent of weight loss. The positive effect of social interaction by participating in a trial may underlay this improvement. However, physical activity has also been described to improve mental health [28,29]. This effect was not observed in men, which may be explained by their better mental health at baseline. Mental health improvements may stimulate compliance to a lifestyle change over time. In our study, an additional questionnaire showed that 66% of the participants indicated that they maintained their new lifestyle one year after the intervention, possibly contributed by a ‘buddy’ effect for these older adult couples.

In conclusion, reducing energy balance by 25% for 13 weeks by a modest change in dietary habits and physical exercise seemed generally feasible in older adults (mean age 63 years) and resulted in a weight change comparable to younger adults. Despite a considerable heterogeneity in response, metabolic health was generally beneficially influenced, as reflected by most markers of body composition, blood pressure, physical functioning, glucose, lipid, and thyroid metabolism, and a range of metabolites that could be measured with a well-standardized ^1^H-NMR assay. We conclude that monitoring of the response to an intervention among elderly is optimized by applying metabolomics assays in addition to clinical markers of metabolic health. The response to the lifestyle intervention applied in GOTO, as measured by metabolomics profiles and parameters of wellbeing, can be used as reference for more specific dietary interventions that the ageing field is planning [30].

## METHODS

### Study population

Participants for the GOTO study were recruited from the Leiden Longevity Study (LLS), a longitudinal cohort consisting of 421 families of long-lived Caucasian siblings, together with their offspring and the partners thereof [31]. For the current intervention couples consisting of offspring from the long-lived siblings and their current partners were included. In case one of the two was not eligible to participate, single offspring or controls were included to obtain the required sample size. Individuals of ages between 46 and 75 years and having a BMI ≥23 ≤35 kg/m^2^ were recruited between February and October 2012. Potential participants underwent a telephonic screening and a screenings home visit. Exclusion criteria were: type I or type II diabetes (on diabetic medication); fasting blood glucose level ≥7.0 mmol/L; weight change ≥3 kg over the past 6 months; engagement in heavy/intensive physical activity (top sport or physically heavy work); any disease or condition that seriously affects body weight and/or body composition including active types of cancer; heart failure (NYHA III/VI), COPD (GOLD III/VI); recent (<3 months prior to intervention) immobilisation for >1 week; psychiatric or behavioural problems; use of thyroid medication, immunosuppressive drugs (e.g. prednisone, methotrexate, biologicals (TNF-alpha antagonists); concurrent participation in any other intervention study or weight management program, or not having a general practitioner. The Medical Ethical Committee of the Leiden University Medical Center approved the study and all participants signed written informed consent. All experiments were performed in accordance with relevant and approved guidelines and regulations. This trial was registered at the Dutch Trial Register (http://www.trialregister.nl) as NTR3499.

### Intervention

The intervention comprised 13 weeks of 25% lowered energy balance by 12.5% reduction in energy intake and 12.5% increase in physical activity. Baseline energy intake and expenditure were assessed by an online version of a 150-item food frequency questionnaire (FFQ) [32] and by the International Physical Activity Questionnaire- Short Form (IPAQ-SF) [33]. The IPAQ-SF collects information on time spent walking, in moderate and vigorous physical activity, and sitting over the last seven days to estimate the total metabolic equivalent (MET) in minutes per week.

Individual guidelines were prescribed by respectively a dietician and physiotherapist in consultation with the participant to match the subjects' preferences and physical capabilities. The dietary guidelines were as much as possible according to the ‘Dutch Guidelines for a healthy diet’ [34]. Participants were advised to increase the amount of physical activity in such a way it fitted in their daily life pattern, as couple or alone, by walking, cycling, adjusted activities in and around the house and participation in local sport activities and facilities.

During the intervention participants had weekly contact with the dietician and physiotherapist by phone, email or at the participants home (alternating schedule) to check and stimulate adherence to the intervention and to discuss practical problems and solutions. To optimally guide the participants, both dietician and physiotherapist combined elements from the Attitude, Social influence and self-Efficacy model (ASE) [35], the Stages of Change Model [36] and Motivational Interviewing [37].

Participants daily recorded their eating behaviour and physical activity in a diary. To quantitatively assess dietary intake two telephonic 24-h recalls were performed during the first month and two during the last month of the intervention. Days of the recall were unannounced to the participant and randomized to obtain a good distribution of the different days of the week, including weekend days. During the monthly home visits body weight and body composition were measured. To quantitatively determine physical activity prior to the intervention and at the end of the intervention accelerometers worn at home during seven days on wrist and ankle (GENEActiv, Activinsights, Kimbolton, UK) were used.

### Anthropometrics

Height and weight were measured to the nearest 0.1 cm and 0.1 kg, respectively (Seca Clara 803, Seca Deutschland, Hamburg, Germany), with the person dressed in light clothing and without shoes. Waist circumference was measured to the nearest cm at the midpoint between the lowest rib and the top of the iliac crest with a non-elastic tape in standing position without shoes. Fat free mass and fat mass were measured using the In-Body 720 body composition analyser (Biospace, Cerritos, CA, USA).

### Blood pressure

Trained staff members measured blood pressure in sitting position after a 10-minute rest on the dominant arm using a validated blood pressure device (Maxi-Stabil 3, Welch Allyn, Leiden, the Netherlands). Blood pressure was measured 4 times, twice in the first part of the afternoon and twice at the end of the afternoon. Systolic and diastolic blood pressures were calculated as the average of the four measurements.

### Energy metabolism

Resting metabolic rate was measured 65 minutes after a standardized meal by indirect calorimetry, using a ventilated hood system (Care Fusion Canopy Jaeger Oxycon Pro, CareFusion Germany, Hoechberg, Germany). The standardized meal was 500 kcal and consisted of one raisin bun, one whole-wheat bun with margarine and 20 grams of Gouda cheese and a cup of tea without milk or sugar. Participants were lying on a bed under the ventilated hood in a quiet, temperature controlled room for 30 minutes. The initial 5 minutes of the measurement were not used for the analysis. VO2 and VCO2 were measured every minute. Resting energy expenditure (REE) and respiratory quotient (RQ) were calculated using the formulas:

REE=3.91 VO2+1.10 VCO2−1.93N.

RQ=VCO2/VO2

To exclude outliers in energy expenditure, the degree of variation based on the coefficient of variation (CV) was examined based on the mean and the SD of five data points that were used. Data per 5 consecutive minutes was included if the RQ data over these 5 minutes had a CV <5%.

### Physical performance

Physical performance was assessed by the short physical performance battery (SPPB), which consisted of three components: balance, gait speed, and chair rise ability [38]. Handgrip strength was determined by three consecutive measures using a hand dynamometer (Jamar, Lafayette Instrument, Lafayette, IN, USA) at both hands.

### Quality of life

Quality of life was assessed using the Short Form Health Survey-12 (SF-12). This questionnaire [39] distinguishes physical and mental health, each assessed by six items.

### Framingham risk score

The Framingham risk score (FRS), which estimates the 10-year risk for developing coronary heart disease, was calculated using the criteria proposed by the Expert Panel on Detection, Evaluation, and Treatment of High Blood Cholesterol in Adults [40]. The score is based on the age, gender, total and HDL cholesterol serum level, smoking status, and systolic blood pressure of an individual.

### Diagnostic measurements

All measurements were performed in fasted serum collected by venipuncture. Cholesterol, free thyroxine (fT4), glucose, high-density lipoprotein (HDL) cholesterol, triglycerides, high-sensitivity C-reactive protein (hsCRP), and thyroid stimulating hormone (TSH) were measured on the Roche/Hitachi Modular P800 analyzer (Roche Diagnostics, Almere, The Netherlands). Dehydro-epiandrosterone sulfate (DHEAS), insulin, insulin-like growth factor 1 (IGF-1), and insulin-like growth factor 1 binding protein 3 (IGF-BP3) were assessed on the Immulite 2000 XPi (Siemens, Eschborn, Germany). Adiponectin and leptin were determined using Human Adiponectin and Leptin RIA kits (EMD Millipore Corporation, Billerica, MA, USA). Free triiodothyronine (fT3) was determined using the ARCHITECT Free T3 assays (Abbott Laboratories, Abbott Park, IL, USA) on the Hitachi Modular E170 analyzer (Roche Diagnostics). Coefficients of variation for all laboratory analyses were <8%. Low-density lipoprotein (LDL) cholesterol was calculated using the Friedewald formula [41], while the homeostasis model assessment-estimated insulin resistance (HOMA-IR) was calculated using the publically available HOMA calculator (https://www.dtu.ox.ac.uk/homacalculator) [42].

### Hydrogen-1 Nuclear Magnetic Resonance metabolites

^1^H-NMR metabolites were measured using a previously described platform [43]. For our analysis we used the total lipid concentrations, fatty acid composition, and low-molecular-weight metabolites, including amino acids, glycolysis-related metabolites, ketone bodies and metabolites involved in fluid balance and immunity. All metabolite concentrations were natural log-transformed and scaled to standard deviation units before analysis.

### Statistical analysis

Baseline differences between longevity family members and controls were calculated using a linear mixed model adjusted for age, gender (fixed effects), and household (random effects). The effects of the intervention were determined using a linear mixed model adjusted for age, gender, status (longevity family member or control) (fixed effects), household, and individual (random effects). Parameters were analyzed separately in men and women if there was a significant gender-difference at baseline. For additional analyses, weight was added to the model to determine weight loss-independent effects.

Principle Component Analyses and the following association analyses with age, gender and intervention were performed in R [44]. Principle Components (PCs) were computed using the function *prcomp* of the *stats* package [44]. Association analyses with the obtained PCs were performed using mixed linear models, function *lmer*, of package *lmerTest* [45]. Heatmaps were drawn to visualize the magnitude of the statistics from the association analyses using *labeledHeatmap* of the *WGCNA* package [46].

Sample size calculation was based on fasting insulin as primary end point, whereby we assumed a decrease of 21% (9.75 to 7.69 μIU/mL), since this was observed after 3 months in the calorie restriction with exercise group of the CALERIE study [6], which is comparable to our study. As the mean fasting insulin levels (μIU/mL) in longevity family members and controls in the complete LLS cohort were 6.93 (SD=4.3) and 8.70 (SD=6.6), respectively, and a correlation of 0.6 between repeated insulin measurements was assumed [47], we based our power calculation on an expected mean decrease of 1.65 μIU/mL, SD 4.9. With a power of 80% and an α of 5% this translates in a required sample size of 72 individuals. In a recent large meta-analysis, genetic background did not influence the association of healthy diet with fasting glucose or insulin [48]. Nevertheless, we doubled the sample size of our study to account for potential differences between longevity family members and controls in response to the intervention. Taking into account a dropout rate of 10%, we aimed to include 80 couples in the intervention.

All statistical analyses were performed with STATA/SE 11.2 (StataCorp LP, College Station, TX, USA) and SPSS Statistics v20 (IBM Corp, Armonk, NY, USA) and a *P*≤0.05 was considered significant.

## SUPPLEMENTARY INFORMATION TABLES AND FIGURES


