# Supplementary material for: Metabolic effects of a 13-weeks lifestyle intervention in older adults: The Growing Old Together Study
Source: Aging (Albany NY). 2016 Jan 25;8(1):111–24. doi: 10.18632/aging.100877 (PMC4761717; doi:10.18632/aging.100877)
Supplement: Supplementary file 1 [file aging-08-111-s001.doc]

**Supplementary Information for**

**Metabolic effects of a 13-weeks lifestyle intervention in older adults: The Growing Old Together Study**

Ondine van de Rest, PhD^#1^, Bianca A.M. Schutte, MSc^#2^, Joris Deelen, PhD^#2^, Stephanie A.M. Stassen, MSc^3^, Erik B. van den Akker, PhD^2,4^, Diana van Heemst, PhD ^3^, Petra Dibbets-Schneider, BSc^5^, Regina. A. van Dipten-van der Veen, BSc^1^, Milou Kelderman, BSc^1^, Thomas Hankemeier, PhD^6^, Simon P. Mooijaart, PhD ^3^, Jeroen van der Grond, PhD ^5^, Jeanine J. Houwing-Duistermaat, PhD ^7^, Marian Beekman, PhD ^2^, Edith J.M. Feskens, PhD ^1^, P. Eline Slagboom, PhD^2^

^#^ These authors contributed equally to this work

1. Division of Human Nutrition, Wageningen University, PO Box 8129, 6700 EV Wageningen, The Netherlands.
2. Department of Molecular Epidemiology, Leiden University Medical Center, PO Box 9600, 2300 RC Leiden, The Netherlands.
3. Department of Gerontology and Geriatrics, Leiden University Medical Center, PO Box 9600, 2300 RC Leiden, The Netherlands
4. The Delft Bioinformatics Lab, Delft University of Technology, Mekelweg 4, 2628 CD Delft, The Netherlands
5. Department of Radiology, Leiden University Medical Center, PO Box 9600, 2300 RC Leiden, The Netherlands
6. Division of Analytical Biosciences, Leiden Academic Centre for Drug Research, Leiden University, Leiden 2300 RA, The Netherlands

**Suplementary Tables**

**Supplementary Table 1A. Baseline characteristics of parameters of body composition, health and functioning, and diagnostic measurements.**

| **Characteristic** | |  | ***n*** | **Longevity family members** | ***n*** | **Controls** | ***P*-value^a^** |
| --- | --- | --- | --- | --- | --- | --- | --- |
|  | Women, n (%) |  |  | 39 (43.3) |  | 42 (56.8) |  |
|  | Age, mean (SD) [range], years |  | 90 | 63.4 (5.4) [49.1-75.1] | 74 | 62.4 (6.1) [46.7-73.5] | 0.24 |
| **Body composition, mean (SD) [range]** | |  |  |  |  |  |  |
|  | Weight, kg |  | 89 | 79.8 (9.6) [62.5-105.7] | 73 | 79.0 (10.2) [60.5-102.4] | 0.74 |
|  |  | Men | 50 | 84.3 (8.0) [67.2-105.7] | 31 | 85.4 (8.1) [70.1-102.4] | 0.39 |
|  |  | Women | 39 | 74.1 (8.4) [62.5-95.4] | 42 | 74.1 (8.9) [60.5-100.4] | 0.73 |
|  | BMI, kg/m^2^ |  | 89 | 27.0 (2.6) [22.9-34.2] | 73 | 26.9 (2.4) [22.9-33.5] | 0.77 |
|  | Waist circumference, cm |  | 90 | 96.2 (7.9) [74-122] | 74 | 96.1 (8.2) [77-112] | 0.47 |
|  |  | Men | 51 | 98.1 (7.4) [80-122] | 32 | 100.1 (6.4) [89-112] | 0.26 |
|  |  | Women | 39 | 93.6 (7.9) [74-112] | 42 | 93.0 (8.1) [77-111] | 0.63 |
|  | Body fat, % |  | 89 | 29.6 (8.8) [11.6-48.3] | 73 | 30.9 (7.0) [17.1-51.0] | 0.98 |
|  |  | Men | 50 | 23.7 (5.7) [11.6-38.7] | 31 | 25.5 (4.7) [17.1-35.0] | 0.17 |
|  |  | Women | 39 | 37.1 (5.8) [22.9-48.3] | 42 | 34.9 (5.6) [21.0-51.0] | 0.13 |
|  | Fat free mass, kg^2^ |  | 89 | 56.3 (10.1) [38.5-78.6] | 74 | 54.6 (10.1) [37.0-81.6] | 0.58 |
|  |  | Men | 50 | 64.1 (5.6) [49.5-78.6] | 31 | 63.6 (7.5) [46.9-81.6] | 0.98 |
|  |  | Women | 39 | 46.3 (3.9) [38.5-54.7] | 42 | 48.0 (5.9) [37.0-65.5] | 0.30 |
| **Health and functioning, mean (SD) [range]** | | |  |  |  |  |  |
|  | Systolic blood pressure, mmHg^b^ |  | 65 | 135.4 (15.9) [111-196] | 48 | 137.8 (17.1) [101-173] | 0.99 |
|  | Diastolic blood pressure, mmHg^b^ |  | 65 | 83.5 (7.4) [64-101] | 48 | 84.7 (9.2) [65-108] | 0.28 |
|  | REE, kcal/day |  | 68 | 1,186 (178) [890-1,680] | 58 | 1,189 (163) [871-1,680] | 0.42 |
|  |  | Men | 38 | 1,294 (150) [940-1,680] | 27 | 1,282 (160) [1,083-1,680] | 0.93 |
|  |  | Women | 30 | 1,048 (96) [890-1,300] | 31 | 1,108 (117) [872-1,349] | **0.04** |
|  | Handgrip strength, kg |  | 89 | 43.2 (12.1) [21-67] | 71 | 40.8 (10.3) [21-78] | 0.82 |
|  |  | Men | 50 | 52.3 (7.5) [36-67] | 30 | 49.5 (7.5) [39-78] | 0.20 |
|  |  | Women | 39 | 31.6 (4.8) [21-42] | 41 | 34.4 (6.6) [21-54] | 0.18 |
|  | Physical functioning |  | 90 | 11.5 (0.6) [10-12] | 73 | 11.6 (0.7) [10-12] | 0.31 |
|  | Physical quality of life |  | 88 | 52.4 (5.9) [26.7-62.8] | 72 | 52.9 (6.2) [26.8-63.0] | 0.61 |
|  |  | Men | 51 | 52.7 (4.8) [39.0-58.9] | 31 | 53.0 (4.2) [37.5-56.8] | 0.72 |
|  |  | Women | 37 | 51.9 (7.2) [26.7-62.9] | 41 | 52.7 (7.5) [26.8-63.0] | 0.78 |
|  | Mental quality of life |  | 88 | 54.5 (6.7) [27.6-63.7] | 72 | 53.6 (8.2) [19.4-67.0] | 0.71 |
|  |  | Men | 51 | 56.2 (4.9) [41.5-63.7] | 31 | 55.6 (5.7) [29.0-62.9] | 0.56 |
|  |  | Women | 37 | 52.3 (8.1) [27.6-60.8] | 41 | 52.1 (9.4) [19.4-67.0] | 0.65 |
|  | FRS, % |  | 90 | 8.3 (5.0) [1-20] | 74 | 8.9 (7.3) [1-25] | **<0.001** |
|  |  | Men | 51 | 11.4 (3.6) [6-20] | 32 | 16.2 (4.6) [10-25] | **<0.001** |
|  |  | Women | 39 | 4.3 (3.4) [1-17] | 42 | 3.3 (2.2) [1-11] | 0.67 |
| **Medication use, *n* (%)** | |  |  |  |  |  |  |
|  | Lipid-lowering agent |  | 90 | 11 (12.2) | 74 | 18 (24.3) | **0.03** |
|  | Antihypertensive agent |  | 90 | 23 (25.6) | 74 | 26 (35.1) | 0.22 |
| **Diagnostic measurements, mean (SD) [range]** | | |  |  |  |  |  |
|  | Fasting glucose, mmol/L |  | 90 | 5.0 (0.5) [3.6-6.5] | 74 | 5.0 (0.6) [4.0-7.6] | 0.88 |
|  | Fasting insulin, mU/L^c^ |  | 90 | 9.4 (5.1) [2.0-29.6] | 74 | 9.0 (3.9) [2.0-22.6] | 0.84 |
|  | HOMA-IR |  | 88 | 1.2 (0.6) [0.4-3.8] | 72 | 1.2 (0.5) [0.4-2.7] | 0.72 |
|  | Total cholesterol, mmol/L^d^ |  | 79 | 5.5 (1.0) [3.3-8.6] | 56 | 5.5 (1.0) [3.2-8.0] | 0.58 |
|  | HDL cholesterol, mmol/L^d^ |  | 79 | 1.6 (0.4) [0.6-3.1] | 56 | 1.4 (0.4) [0.6-2.3] | **0.001** |
|  |  | Men | 43 | 1.4 (0.3) [1.0-2.0] | 23 | 1.1 (0.2) [0.6-1.6] | **<0.001** |
|  |  | Women | 36 | 1.7 (0.5) [0.6-3.1] | 33 | 1.6 (0.3) [1.2-2.3] | 0.39 |
|  | LDL cholesterol, mmol/L^d^ |  | 79 | 3.5 (0.8) [1.8-6.4] | 56 | 3.4 (0.9) [1.6-6.0] | 0.51 |
|  | Triglycerides, mmol/L^c,d^ |  | 79 | 1.3 (0.5) [0.6-3.0] | 56 | 1.2 (0.6) [0.5-4.1] | 0.13 |
|  | fT3, pmol/L |  | 90 | 4.5 (0.4) [3.3-5.5] | 74 | 4.6 (0.4) [3.5-6.2] | 0.54 |
|  | fT4, pmol/L |  | 90 | 15.0 (1.8) [10.5-20.3] | 74 | 14.7 (1.6) [10.5-19.3] | 0.21 |
|  | TSH, mU/L^c^ |  | 90 | 2.6 (1.6) [0.3-9.8] | 74 | 3.1 (3.0) [0.2-25.0] | 0.32 |
|  | DHEAS, nmol/L^c^ |  | 90 | 2.9 (1.8) [0.5-8.6] | 74 | 3.3 (1.6) [0.7-6.8] | **0.006** |
|  |  | Men | 51 | 3.6 (2.0) [0.6-8.6] | 32 | 3.8 (1.4) [1.5-6.8] | 0.12 |
|  |  | Women | 39 | 2.0 (1.2) [0.5-5.2] | 42 | 2.8 (1.6) [0.7-6.7] | **0.03** |
|  | Leptin, µg/L^c^ |  | 90 | 13.5 (11.5) [1.2-61.7] | 74 | 14.4 (9.2) [2.3-50.3] | 0.52 |
|  |  | Men | 51 | 6.7 (3.8) [1.2-17.2] | 32 | 7.6 (3.5) [2.3-18.1] | 0.15 |
|  |  | Women | 39 | 22.5 (12.2) [4.5-61.7] | 42 | 19.7 (8.9) [6.5-50.3] | 0.35 |
|  | Adiponectin, mg/L^c^ |  | 90 | 10.6 (5.2) [2.2-28.1] | 74 | 10.1 (5.5) [2.7-25.6] | **0.04** |
|  |  | Men | 51 | 8.1 (3.5) [2.2-19.3] | 32 | 6.8 (3.0) [2.8-15.2] | 0.08 |
|  |  | Women | 39 | 13.9 (5.2) [5.6-28.1] | 42 | 12.7 (5.6) [2.7-25.6] | 0.30 |
|  | IGF-1, nmol/L |  | 90 | 20.0 (4.8) [7.8-31.9] | 74 | 20.0 (4.8) [8.4-43.0] | 0.57 |
|  |  | Men | 51 | 20.8 (4.7) [7.8-31.9] | 32 | 21.6 (5.1) [10.7-31.7] | 0.41 |
|  |  | Women | 39 | 19.0 (4.6) [11.4-30.9] | 42 | 19.3 (6.4) [8.4-43.0] | 0.88 |
|  | IGFBP-3, mg/L |  | 90 | 4.0 (0.9) [1.7-7.7] | 74 | 4.0 (0.9) [2.2-6.7] | 0.34 |
|  | IGF-1:IGFBP-3 |  | 90 | 0.14 (0.03) [0.06-0.26] | 74 | 0.14 (0.03) [0.07-0.23] | 0.23 |
|  |  | Men | 51 | 0.15 (0.03) [0.07-0.26] | 32 | 0.15 (0.04) [0.10-0.23] | 0.68 |
|  |  | Women | 39 | 0.12 (0.02) [0.06-0.17] | 42 | 0.13 (0.03) [0.07-0.20] | 0.33 |
|  | CRP (high-sensitivity), mg/L^c^ |  | 90 | 1.9 (2.8) [0.2-18.8] | 74 | 2.3 (3.6) [0.2-20.0] | 0.23 |

^a^ P value refers to difference between longevity family members and controls. ^b^ Individuals using antihypertensive agents were removed before analysis. ^c^ Natural log transformed parameter was used for analysis. ^d^ Individuals using lipid-lowering agents were removed before analysis. Parameters were analysed separately in men and women if there was a significant gender-difference at baseline. BMI, body mass index; REE, resting energy expenditure; FRS, Framingham risk score; HOMA-IR, homeostatic model assessment - insulin resistance; HDL, high density lipoprotein; LDL, low density lipoprotein; fT3, free triiodothyronine; fT4, free thyroxine; TSH, thyroid stimulating hormone; DHEAS, dehydroepiandrosterone-sulfate; IGF-1, insuline-like growth factor 1; IGFBP-3, insulin-like growth factor binding protein 3; CRP, C-reactive protein..

**Supplementary Table 1B. Baseline characteristics of ^1^H-NMR metabolites.**

| **Characteristic, mean (SD) [range]** | |  | ***n*** | **Longevity family members** | ***n*** | **Controls** | ***P*-value^a^** |
| --- | --- | --- | --- | --- | --- | --- | --- |
| **Amino acids** | |  |  |  |  |  |  |
|  | Alanine, mmol/L |  | 90 | 0.45 (0.05) [0.34-0.62] | 72 | 0.46 (0.06) [0.33-0.63] | 0.64 |
|  |  | Men | 51 | 0.44 (0.05) [0.35-0.58] | 30 | 0.43 (0.05) [0.33-0.56] | 0.52 |
|  |  | Women | 39 | 0.46 (0.06) [0.34-0.62] | 42 | 0.47 (0.06) [0.36-0.63] | 0.33 |
|  | Glutamine, mmol/L |  | 90 | 0.52 (0.06) [0.39-0.74] | 73 | 0.49 (0.05) [0.36-0.62] | **0.01** |
|  | Glycine, mmol/L |  | 90 | 0.29 (0.06) [0.20-0.56] | 73 | 0.29 (0.06) [0.16-0.54] | 0.30 |
|  |  | Men | 51 | 0.27 (0.04) [0.20-0.40] | 31 | 0.26 (0.04) [0.16-0.39] | 0.15 |
|  |  | Women | 39 | 0.32 (0.07) [0.24-0.56] | 42 | 0.32 (0.07) [0.21-0.54] | 0.94 |
|  | Histidine, mmol/L |  | 90 | 0.06 (0.01) [0.04-0.09] | 73 | 0.06 (0.01) [0.03-0.08] | 0.06 |
| *Branched-chain amino acids* | |  |  |  |  |  |  |
|  | Isoleucine, mmol/L |  | 90 | 0.06 (0.02) [0.04-0.14] | 72 | 0.06 (0.02) [0.03-0.11] | 0.40 |
|  |  | Men | 51 | 0.06 (0.01) [0.04-0.10] | 30 | 0.07 (0.01) [0.05-0.11] | 0.09 |
|  |  | Women | 39 | 0.06 (0.02) [0.04-0.14] | 42 | 0.06 (0.01) [0.03-0.11] | 0.63 |
|  | Leucine, mmol/L |  | 90 | 0.09 (0.01) [0.05-0.13] | 72 | 0.09 (0.01) [0.06-0.14] | 0.64 |
|  |  | Men | 51 | 0.09 (0.01) [0.07-0.12] | 30 | 0.09 (0.01) [0.07-0.12] | 0.46 |
|  |  | Women | 39 | 0.08 (0.01) [0.05-0.13] | 42 | 0.08 (0.01) [0.06-0.14] | 0.78 |
|  | Valine, mmol/L |  | 89 | 0.20 (0.03) [0.12-0.28] | 72 | 0.20 (0.03) [0.15-0.34] | 0.44 |
| *Aromatic amino acids* | |  |  |  |  |  |  |
|  | Phenylalanine, mmol/L |  | 90 | 0.08 (0.01) [0.06-0.11] | 73 | 0.08 (0.01) [0.04-0.10] | 0.72 |
|  | Tyrosine, mmol/L |  | 90 | 0.06 (0.01) [0.04-0.08] | 73 | 0.06 (0.01) [0.02-0.09] | 0.48 |
| **Glycolysis-related metabolites** | |  |  |  |  |  |  |
|  | Glucose, mmol/L |  | 90 | 4.26 (0.48) [3.02-6.51] | 73 | 4.22 (0.50) [2.73-6.56] | 0.87 |
|  | Lactate, mmol/L |  | 90 | 1.84 (0.33) [1.22-3.02] | 73 | 1.84 (0.37) [0.71-2.78] | 0.89 |
|  | Pyruvate, mmol/L |  | 89 | 0.08 (0.03) [0.04-0.17] | 73 | 0.09 (0.02) [0.04-0.17] | 0.46 |
|  | Citrate, mmol/L |  | 90 | 0.10 (0.02) [0.06-0.15] | 73 | 0.10 (0.02) [0.04-0.18] | 0.12 |
|  | Glycerol, mmol/L |  | 88 | 0.08 (0.03) [0.04-0.20] | 73 | 0.08 (0.02) [0.03-0.14] | 0.41 |
|  |  | Men | 49 | 0.07 (0.03) [0.04-0.20] | 31 | 0.07 (0.02) [0.04-0.12] | 0.62 |
|  |  | Women | 39 | 0.09 (0.03) [0.04-0.15] | 42 | 0.08 (0.02) [0.03-0.14] | 0.09 |
| **Ketone bodies** | |  |  |  |  |  |  |
|  | Acetate, mmol/L |  | 90 | 0.05 (0.09) [0.03-0.77] | 73 | 0.04 (0.01) [0.02-0.07] | 0.11 |
|  | Acetoacetate, mmol/L |  | 90 | 0.04 (0.02) [0.01-0.15] | 73 | 0.03 (0.01) [0.00-0.10] | 0.59 |
|  |  | Men | 51 | 0.04 (0.02) [0.02-0.15] | 31 | 0.04 (0.02) [0.00-0.10] | 0.50 |
|  |  | Women | 39 | 0.03 (0.01) [0.01-0.07] | 42 | 0.03 (0.01) [0.01-0.07] | 0.87 |
|  | 3-hydroxybutyrate, mmol/L |  | 88 | 0.09 (0.02) [0.04-0.16] | 73 | 0.09 (0.03) [0.03-0.19] | 0.88 |
| **Fatty acids**^b^ | |  |  |  |  |  |  |
|  | Total fatty acids, mmol/L |  | 76 | 12.11 (2.46) [8.55-23.03] | 55 | 12.42 (2.02) [8.70-17.64] | 0.48 |
|  | Omega-3 fatty acids, mmol/L |  | 76 | 0.44 (0.11) [0.20-0.72] | 55 | 0.46 (0.11) [0.28-0.69] | 0.64 |
|  | Docosahexaenoic acid (DHA), mmol/L |  | 76 | 0.16 (0.05) [0.06-0.27] | 55 | 0.16 (0.04) [0.10-0.26] | 0.77 |
|  | Omega-6 fatty acids, mmol/L |  | 76 | 4.18 (0.72) [3.09-6.21] | 55 | 4.25 (0.65) [3.02-6.34] | 0.74 |
|  | Linoleic acid, mmol/L |  | 76 | 3.37 (0.58) [2.49-5.20] | 55 | 3.44 (0.55) [2.36-5.13] | 0.66 |
|  | Polyunsaturated fatty acids, mmol/L |  | 76 | 4.62 (0.79) [3.49-6.83] | 55 | 4.71 (0.71) [3.42-7.03] | 0.72 |
|  | Monounsaturated fatty acids, mmol/L |  | 76 | 2.76 (0.83) [1.54-6.49] | 55 | 2.85 (0.68) [1.80-5.35] | 0.35 |
|  | Saturated fatty acids, mmol/L |  | 76 | 4.73 (0.99) [3.28-9.71] | 55 | 4.87 (0.84) [3.35-6.93] | 0.46 |
|  | Fatty acid chain length |  | 76 | 17.32 (0.34) [16.74-18.50] | 55 | 17.38 (0.31) [16.66-18.20] | 0.46 |
| **Fatty acid ratios, relative to total fatty acids^b^** | |  |  |  |  |  |  |
|  | Omega-3 fatty acids, % |  | 76 | 3.68 (0.64) [2.28-5.47] | 55 | 3.71 (0.77) [2.15-5.77] | 0.65 |
|  | Docosahexaenoic acid (DHA), % |  | 76 | 1.29 (0.31) [0.73-2.33] | 55 | 1.30 (0.32) [0.62-2.03] | 0.57 |
|  | Omega-6 fatty acids, % |  | 76 | 34.75 (2.86) [26.55-41.19] | 55 | 34.38 (2.36) [28.29-39.22] | 0.36 |
|  | Linoleic acid, % |  | 76 | 28.08 (2.69) [20.56-35.62] | 55 | 27.84 (2.40) [21.81-34.00] | 0.57 |
|  | Polyunsaturated fatty acids, % |  | 76 | 38.43 (2.88) [29.65-43.47] | 55 | 38.08 (2.59) [30.43-43.80] | 0.33 |
|  | Monounsaturated fatty acids, % |  | 76 | 22.48 (2.60) [17.82-30.75] | 55 | 22.77 (2.78) [18.89-30.91] | 0.25 |
|  | Saturated fatty acids, % |  | 76 | 39.09 (1.75) [35.45-43.55] | 55 | 39.15 (1.82) [35.19-43.52] | 0.97 |
|  | Unsaturation degree, double bonds per fatty acids |  | 76 | 1.19 (0.05) [1.03-1.29] | 55 | 1.19 (0.05) [1.02-1.31] | 0.57 |
| **Fluid balance** | |  |  |  |  |  |  |
|  | Creatinine, mmol/L |  | 90 | 0.06 (0.01) [0.03-0.09] | 73 | 0.06 (0.01) [0.04-0.12] | 0.18 |
|  |  | Men | 51 | 0.07 (0.01) [0.03-0.09] | 31 | 0.07 (0.01) [0.04-0.12] | 0.09 |
|  |  | Women | 39 | 0.05 (0.01) [0.04-0.07] | 42 | 0.05 (0.01) [0.04-0.07] | 0.90 |
|  | Albumin, signal area |  | 90 | 0.09 (0.00) [0.08-0.10] | 73 | 0.09 (0.00) [0.08-0.10] | 0.91 |
| **Inflammation** | |  |  |  |  |  |  |
|  | Glycoprotein acetyls, mainly a1-acid glycoprotein, mmol/L |  | 90 | 1.21 (0.20) [0.91-2.15] | 73 | 1.25 (0.18) [0.88-1.90] | 0.11 |
|  |  | Men | 51 | 1.17 (0.15) [0.91-1.54] | 31 | 1.31 (0.18) [0.99-1.90] | **<0.001** |
|  |  | Women | 39 | 1.26 (0.24) [0.97-2.15] | 42 | 1.20 (0.17) [0.88-1.57] | 0.25 |
| **Apolipoproteins^b^** | |  |  |  |  |  |  |
|  | Apolipoprotein B, g/L |  | 79 | 0.94 (0.23) [0.56-1.94] | 55 | 0.99 (0.18) [0.61-1.51] | 0.07 |
|  | Apolipoprotein A1, g/L |  | 79 | 1.60 (0.17) [1.21-2.07] | 55 | 1.55 (0.21) [1.08-2.10] | **<0.001** |
|  |  | Men | 43 | 1.53 (0.15) [1.21-1.87] | 22 | 1.39 (0.13) [1.08-1.57] | **<0.001** |
|  |  | Women | 36 | 1.68 (0.15) [1.43-2.07] | 33 | 1.66 (0.18) [1.28-2.10] | 0.68 |
| **Lipids^b^** |  |  |  |  |  |  |  |
|  | Triglycerides, mmol/L |  | 79 | 1.16 (0.65) [0.41-4.88] | 55 | 1.26 (0.50) [0.63-3.16] | **0.03** |
|  |  | Men | 43 | 1.13 (0.40) [0.59-2.09] | 22 | 1.55 (0.56) [0.81-3.16] | **<0.001** |
|  |  | Women | 36 | 1.20 (0.86) [0.41-4.88] | 33 | 1.07 (0.35) [0.63-2.02] | 0.88 |
|  | Phosphoglycerides, mmol/L |  | 76 | 2.14 (0.32) [1.54-3.01] | 55 | 2.16 (0.37) [1.43-2.92] | 0.55 |
|  |  | Men | 42 | 2.06 (0.30) [1.54-2.62] | 22 | 1.97 (0.30) [1.43-2.45] | 0.33 |
|  |  | Women | 34 | 2.24 (0.32) [1.69-3.01] | 33 | 2.29 (0.36) [1.60-2.92] | 0.55 |
|  | Cholines, mmol/L |  | 76 | 2.55 (0.33) [1.93-3.30] | 55 | 2.57 (0.40) [1.68-3.42] | 0.45 |
|  |  | Men | 42 | 2.45 (0.34) [1.93-3.18] | 22 | 2.35 (0.32) [1.68-2.91] | 0.32 |
|  |  | Women | 34 | 2.67 (0.29) [2.07-3.30] | 33 | 2.71 (0.38) [2.00-3.42] | 0.61 |
|  | Sphingomyelins, mmol/L |  | 76 | 0.51 (0.09) [0.33-0.79] | 55 | 0.51 (0.08) [0.32-0.69] | 0.22 |
|  |  | Men | 42 | 0.48 (0.09) [0.33-0.79] | 22 | 0.47 (0.07) [0.32-0.62] | 0.55 |
|  |  | Women | 34 | 0.55 (0.07) [0.41-0.71] | 33 | 0.54 (0.08) [0.39-0.69] | 0.81 |
| **Total lipid concentrations in lipoprotein subclasses^b^** | |  |  |  |  |  |  |
|  | Extremely large VLDL, mmol/L |  | 73 | 0.03 (0.02) [0.00-0.14] | 52 | 0.03 (0.02) [0.00-0.11] | 0.12 |
|  | Very large VLDL, mmol/L |  | 68 | 0.06 (0.07) [0.00-0.41] | 49 | 0.06 (0.06) [0.01-0.31] | 0.08 |
|  | Large VLDL, mmol/L |  | 73 | 0.22 (0.23) [0.02-1.56] | 53 | 0.23 (0.20) [0.03-1.09] | 0.42 |
|  | Medium VLDL, mmol/L |  | 79 | 0.48 (0.37) [0.09-2.64] | 55 | 0.52 (0.30) [0.17-1.67] | 0.06 |
|  | Small VLDL, mmol/L |  | 79 | 0.61 (0.26) [0.16-1.90] | 55 | 0.67 (0.20) [0.31-1.06] | **0.01** |
|  | Very small VLDL, mmol/L |  | 79 | 0.58 (0.16) [0.24-1.06] | 55 | 0.63 (0.14) [0.34-1.01] | **0.03** |
|  | IDL, mmol/L |  | 79 | 1.28 (0.30) [0.76-2.22] | 55 | 1.35 (0.28) [0.77-2.24] | 0.14 |
|  | Large LDL, mmol/L |  | 79 | 1.47 (0.35) [0.87-2.53] | 55 | 1.55 (0.33) [0.82-2.57] | 0.19 |
|  | Medium LDL, mmol/L |  | 79 | 0.84 (0.21) [0.46-1.43] | 55 | 0.88 (0.19) [0.44-1.47] | 0.20 |
|  | Small LDL, mmol/L |  | 79 | 0.54 (0.13) [0.31-0.89] | 55 | 0.56 (0.12) [0.30-0.93] | 0.30 |
|  | Very large HDL, mmol/L |  | 79 | 0.48 (0.21) [0.10-1.14] | 55 | 0.42 (0.21) [0.11-0.96] | **0.001** |
|  |  | Men | 43 | 0.41 (0.16) [0.10-0.92] | 22 | 0.26 (0.10) [0.11-0.56] | **<0.001** |
|  |  | Women | 36 | 0.55 (0.23) [0.13-1.14] | 33 | 0.53 (0.20) [0.18-0.96] | 0.95 |
|  | Large HDL, mmol/L |  | 75 | 0.80 (0.29) [0.25-1.73] | 51 | 0.73 (0.34) [0.23-1.55] | **<0.001** |
|  |  | Men | 41 | 0.68 (0.24) [0.25-1.15] | 18 | 0.45 (0.16) [0.23-0.83] | **<0.001** |
|  |  | Women | 34 | 0.95 (0.29) [0.54-1.73] | 33 | 0.89 (0.31) [0.34-1.55] | 0.29 |
|  | Medium HDL, mmol/L |  | 79 | 0.95 (0.16) [0.66-1.44] | 55 | 0.93 (0.20) [0.51-1.38] | **0.005** |
|  |  | Men | 43 | 0.91 (0.15) [0.66-1.44] | 22 | 0.79 (0.15) [0.51-1.10] | **0.003** |
|  |  | Women | 36 | 1.01 (0.15) [0.70-1.30] | 33 | 1.02 (0.17) [0.72-1.38] | 0.88 |
|  | Small HDL, mmol/L |  | 79 | 1.15 (0.11) [0.92-1.42] | 55 | 1.15 (0.10) [0.95-1.38] | 0.70 |
|  |  | Men | 43 | 1.15 (0.10) [0.92-1.41] | 22 | 1.12 (0.09) [0.95-1.31] | 0.30 |
|  |  | Women | 36 | 1.15 (0.13) [0.95-1.42] | 33 | 1.16 (0.10) [0.97-1.38] | 0.48 |
| **Lipoprotein particle size^b^** | |  |  |  |  |  |  |
|  | VLDL diameter, nm |  | 79 | 35.76 (1.11) [33.87-39.22] | 55 | 35.87 (1.26) [34.26-39.69] | 0.28 |
|  | LDL diameter, nm |  | 79 | 23.58 (0.11) [23.33-23.83] | 55 | 23.60 (0.10) [23.43-23.81] | 0.30 |
|  | HDL diameter, nm |  | 79 | 9.97 (0.24) [9.46-10.61] | 55 | 9.90 (0.27) [9.39-10.47] | **0.001** |
|  |  | Men | 43 | 9.90 (0.20) [9.46-10.41] | 22 | 9.67 (0.17) [9.39-10.08] | **<0.001** |
|  |  | Women | 36 | 10.07 (0.25) [9.48-10.61] | 33 | 10.04 (0.22) [9.64-10.47] | 0.62 |
| **Cholesterol^b^** | |  |  |  |  |  |  |
|  | Total cholesterol, mmol/L |  | 79 | 5.05 (0.90) [3.64-7.70] | 55 | 5.16 (0.91) [2.98-7.95] | 0.77 |
|  | VLDL cholesterol, mmol/L |  | 79 | 0.76 (0.33) [0.22-2.27] | 55 | 0.83 (0.24) [0.36-1.38] | **0.02** |
|  | IDL cholesterol, mmol/L |  | 79 | 0.82 (0.20) [0.48-1.47] | 55 | 0.86 (0.19) [0.45-1.48] | 0.23 |
|  | LDL cholesterol, mmol/L |  | 79 | 1.93 (0.51) [1.04-3.45] | 55 | 2.02 (0.47) [0.98-3.52] | 0.22 |
|  | HDL cholesterol, mmol/L |  | 79 | 1.55 (0.32) [0.79-2.49] | 55 | 1.45 (0.38) [0.68-2.35] | **<0.001** |
|  |  | Men | 43 | 1.44 (0.25) [0.96-1.96] | 22 | 1.14 (0.22) [0.68-1.51] | **<0.001** |
|  |  | Women | 36 | 1.68 (0.35) [0.79-2.49] | 33 | 1.66 (0.31) [1.08-2.35] | 0.97 |
|  | HDL2 cholesterol, mmol/L |  | 79 | 1.04 (0.30) [0.28-1.94] | 55 | 0.94 (0.34) [0.25-1.77] | **<0.001** |
|  |  | Men | 43 | 0.94 (0.24) [0.48-1.44] | 22 | 0.66 (0.20) [0.25-0.98] | **<0.001** |
|  |  | Women | 36 | 1.16 (0.32) [0.28-1.94] | 33 | 1.13 (0.28) [0.61-1.77] | 0.99 |
|  | HDL3 cholesterol, mmol/L |  | 79 | 0.51 (0.03) [0.42-0.60] | 55 | 0.51 (0.04) [0.42-0.59] | **0.03** |
|  |  | Men | 43 | 0.51 (0.03) [0.43-0.60] | 22 | 0.48 (0.03) [0.42-0.55] | **0.001** |
|  |  | Women | 36 | 0.52 (0.04) [0.42-0.60] | 33 | 0.53 (0.03) [0.47-0.59] | 0.33 |

^a^ P value refers to difference between longevity family members and controls. ^b^ Individuals using lipid-lowering agents were removed before analysis. All metabolite concentrations were natural log-transformed and scaled to standard deviation units before analysis. Parameters were analysed separately in men and women if there was a significant gender-difference at baseline. VLDL, very low density lipoprotein; IDL, intermediate density lipoprotein; LDL, low density lipoprotein; HDL, high density lipoprotein.

**Supplementary Table 2A. Effects of the intervention on parameters of body composition, health and functioning, and diagnostic measurements.**

|  |  |  |  | **Unadjusted** | | **Weight loss adjusted** | |
| --- | --- | --- | --- | --- | --- | --- | --- |
| **Characteristic, mean (SE)** | |  | ***n*** | **Difference** | ***P*-value^a^** | **Difference** | ***P*-value^b^** |
| **Body composition** | |  |  |  |  |  |  |
|  | Weight, kg |  | 161 | -3.34 (0.18) | **<0.001** |  |  |
|  |  | Men | 80 | -3.42 (0.27) | **<0.001** |  |  |
|  |  | Women | 81 | -3.25 (0.23) | **<0.001** |  |  |
|  | BMI, kg/m^2^ |  | 161 | -1.13 (0.06) | **<0.001** |  |  |
|  | Waist circumference, cm |  | 163 | -4.3 (0.4) | **<0.001** |  |  |
|  |  | Men | 82 | -4.4 (0.6) | **<0.001** |  |  |
|  |  | Women | 81 | -4.2 (0.6) | **<0.001** |  |  |
|  | Body fat, % |  | 161 | -2.26 (0.16) | **<0.001** |  |  |
|  |  | Men | 80 | -2.22 (0.23) | **<0.001** |  |  |
|  |  | Women | 81 | -2.29 (0.21) | **<0.001** |  |  |
|  | Fat free mass, kg^2^ |  | 161 | -0.67 (0.10) | **<0.001** |  |  |
|  |  | Men | 80 | -0.83 (0.16) | **<0.001** |  |  |
|  |  | Women | 81 | -0.51 (0.13) | **<0.001** |  |  |
| **Health and functioning** | |  |  |  |  |  |  |
|  | Systolic blood pressure, mmHg^c^ |  | 113 | -4.33 (0.98) | **<0.001** | -2.74 (1.12) | **0.01** |
|  | Diastolic blood pressure, mmHg^c^ |  | 113 | -1.66 (0.61) | **0.007** | -0.52 (0.68) | 0.44 |
|  | REE, kcal/day |  | 126 | -49.2 (8.0) | **<0.001** | -20.2 (8.3) | **0.02** |
|  |  | Men | 65 | -46.59 (11.76) | **<0.001** | -8.09 (12.41) | 0.51 |
|  |  | Women | 61 | -51.94 (10.79) | **<0.001** | -28.42 (11.28) | **0.01** |
|  | Handgrip strength, kg |  | 153 | 0.38 (0.32) | 0.25 |  |  |
|  |  | Men | 76 | 0.24 (0.53) | 0.65 |  |  |
|  |  | Women | 77 | 0.51 (0.38) | 0.18 |  |  |
|  | Physical functioning |  | 159 | 0.14 (0.05) | **0.01** | 0.11 (0.06) | **0.04** |
|  | Physical quality of life |  | 157 | -0.18 (0.61) | 0.77 |  |  |
|  |  | Men | 82 | -0.72 (0.83) | 0.39 |  |  |
|  |  | Women | 75 | 0.42 (0.92) | 0.65 |  |  |
|  | Mental quality of life |  | 157 | 0.9 (0.70) | 0.19 |  |  |
|  |  | Men | 82 | -1.13 (0.84) | 0.18 |  |  |
|  |  | Women | 75 | 3.13 (1.12) | **0.01** | 3.13 (1.16) | **0.007** |
|  | FRS, % |  | 163 | -0.51 (0.23) | **0.03** | -0.09 (0.24) | 0.73 |
|  |  | Men | 82 | -0.65 (0.43) | 0.13 |  |  |
|  |  | Women | 81 | -0.37 (0.15) | **0.01** | -0.15 (0.17) | 0.38 |
| **Diagnostic measurements** | |  |  |  |  |  |  |
|  | Fasting glucose, mmol/L |  | 163 | -0.06 (0.04) | 0.16 |  |  |
|  | Fasting insulin, mU/L^d^ |  | 163 | -0.05 (0.03) | **0.04** | 0.04 (0.03) | 0.21 |
|  | HOMA-IR |  | 153 | -0.03 (0.03) | 0.33 |  |  |
|  | Total cholesterol, mmol/L^e^ |  | 135 | -0.29 (0.06) | **<0.001** | -0.28 (0.06) | **<0.001** |
|  | HDL cholesterol, mmol/L^e^ |  | 135 | -0.01 (0.02) | 0.49 |  |  |
|  |  | Men | 66 | 0.04 (0.02) | 0.11 |  |  |
|  |  | Women | 69 | -0.06 (0.03) | **0.02** | -0.08 (0.03) | **0.02** |
|  | LDL cholesterol, mmol/L^e^ |  | 135 | -0.26 (0.05) | **<0.001** | -0.23 (0.05) | **<0.001** |
|  | Triglycerides, mmol/L^d,e^ |  | 135 | -0.04 (0.03) | 0.11 |  |  |
|  | fT3, pmol/L |  | 163 | -0.14 (0.03) | **<0.001** | -0.12 (0.03) | **<0.001** |
|  | fT4, pmol/L |  | 163 | -0.07 (0.09) | 0.44 |  |  |
|  | TSH, mU/L^e^ |  | 163 | -0.04 (0.03) | 0.17 |  |  |
|  | DHEAS, nmol/L^d^ |  | 163 | -0.02 (0.01) | 0.20 |  |  |
|  |  | Men | 82 | -0.01 (0.02) | 0.47 |  |  |
|  |  | Women | 81 | -0.02 (0.02) | 0.28 |  |  |
|  | Leptin, µg/L^d^ |  | 163 | -0.26 (0.03) | **<0.001** | -0.11 (0.03) | **<0.001** |
|  |  | Men | 82 | -0.29 (0.04) | **<0.001** | -0.14 (0.05) | **0.002** |
|  |  | Women | 81 | -0.23 (0.03) | **<0.001** | -0.09 (0.03) | **0.009** |
|  | Adiponectin, mg/L^d^ |  | 163 | 0.04 (0.01) | **0.005** | 0.01 (0.02) | 0.78 |
|  |  | Men | 82 | 0.09 (0.02) | **<0.001** | 0.04 (0.03) | 0.11 |
|  |  | Women | 81 | -0.01 (0.02) | 0.76 |  |  |
|  | IGF-1, nmol/L |  | 163 | 0.10 (0.24) | 0.67 |  |  |
|  |  | Men | 82 | 0.36 (0.31) | 0.24 |  |  |
|  |  | Women | 81 | -0.17 (0.35) | 0.64 |  |  |
|  | IGFBP-3, mg/L |  | 163 | -0.05 (0.05) | 0.37 |  |  |
|  | IGF-1:IGFBP-3 |  | 163 | 0.004 (0.003) | 0.21 |  |  |
|  |  | Men | 82 | 0.009 (0.006) | 0.14 |  |  |
|  |  | Women | 81 | -0.001 (0.003) | 0.82 |  |  |
|  | CRP (high-sensitivity), mg/L^d^ |  | 163 | -0.11 (0.07) | 0.09 |  |  |

^a^ P value refers to difference between baseline and end. ^b^ P value refers to difference between baseline and end after adjustement for weight loss. ^c^ Individuals using antihypertensive agents were removed before analysis. ^d^ Natural log transformed parameter was used for analysis. ^e^ Individuals using lipid-lowering agents were removed before analysis. Parameters were analysed separately in men and women if there was a significant gender-difference at baseline. BMI, body mass index; REE, resting energy expenditure; FRS, Framingham risk score; HOMA-IR, homeostatic model assessment - insulin resistance; HDL, high density lipoprotein; LDL, low density lipoprotein; fT3, free triiodothyronine; fT4, free thyroxine; TSH, thyroid stimulating hormone; DHEAS, dehydroepiandrosterone-sulfate; IGF-1, insuline-like growth factor 1; IGFBP-3, insulin-like growth factor binding protein 3; CRP, C-reactive protein.

**Supplementary Table 2B. Effects of the intervention on ^1^H-NMR metabolites.**

|  |  |  |  | **Unadjusted** | | **Weight loss adjusted** | |
| --- | --- | --- | --- | --- | --- | --- | --- |
| **Characteristic, mean (SE)** | |  | ***n*** | **Difference** | ***P*-value^a^** | **Difference** | ***P*-value^b^** |
| **Amino acids** | |  |  |  |  |  |  |
|  | Alanine, mmol/L |  | 161 | 0.011 (0.070) | 0.87 |  |  |
|  |  | Men | 80 | 0.011 (0.101) | 0.92 |  |  |
|  |  | Women | 81 | 0.012 (0.097) | 0.90 |  |  |
|  | Glutamine, mmol/L |  | 162 | 0.194 (0.068) | **0.005** | 0.184 (0.074) | **0.01** |
|  | Glycine, mmol/L |  | 161 | 0.204 (0.042) | **<0.001** |  |  |
|  |  | Men | 81 | 0.253 (0.058) | **<0.001** | 0.234 (0.067) | **0.001** |
|  |  | Women | 80 | 0.153 (0.061) | **0.01** | 0.113 (0.073) | 0.12 |
|  | Histidine, mmol/L |  | 162 | -0.524 (0.095) | **<0.001** | -0.517 (0.099) | **<0.001** |
| *Branched-chain amino acids* | |  |  |  |  |  |  |
|  | Isoleucine, mmol/L |  | 161 | -0.119 (0.062) | 0.05 |  |  |
|  |  | Men | 80 | -0.085 (0.088) | 0.33 |  |  |
|  |  | Women | 81 | -0.152 (0.086) | 0.08 |  |  |
|  | Leucine, mmol/L |  | 161 | -0.178 (0.068) | **0.008** |  |  |
|  |  | Men | 80 | -0.145 (0.089) | 0.10 |  |  |
|  |  | Women | 81 | -0.210 (0.102) | **0.04** | -0.121 (0.107) | 0.26 |
|  | Valine, mmol/L |  | 160 | -0.117 (0.073) | 0.11 |  |  |
| *Aromatic amino acids* | |  |  |  |  |  |  |
|  | Phenylalanine, mmol/L |  | 162 | -0.015 (0.079) | 0.85 |  |  |
|  | Tyrosine, mmol/L |  | 162 | -0.242 (0.087) | **0.005** | -0.163 (0.090) | **0.07** |
| **Glycolysis-related metabolites** | |  |  |  |  |  |  |
|  | Glucose, mmol/L |  | 162 | -0.241 (0.069) | **0.001** | -0.185 (0.075) | **0.01** |
|  | Lactate, mmol/L |  | 162 | -0.049 (0.094) | 0.60 |  |  |
|  | Pyruvate, mmol/L |  | 161 | -0.261 (0.086) | **0.002** | -0.260 (0.090) | **0.004** |
|  | Citrate, mmol/L |  | 162 | 0.199 (0.078) | **0.01** | 0.220 (0.082) | **0.007** |
|  | Glycerol, mmol/L |  | 157 | -0.172 (0.068) | **0.01** |  |  |
|  |  | Men | 77 | -0.178 (0.084) | **0.03** | -0.107 (0.094) | 0.25 |
|  |  | Women | 80 | -0.167 (0.106) | 0.12 |  |  |
| **Ketone bodies** | |  |  |  |  |  |  |
|  | Acetate, mmol/L |  | 162 | -0.012 (0.061) | 0.85 |  |  |
|  | Acetoacetate, mmol/L |  | 162 | -0.097 (0.083) | 0.24 |  |  |
|  |  | Men | 81 | -0.016 (0.122) | 0.90 |  |  |
|  |  | Women | 81 | -0.179 (0.113) | 0.11 |  |  |
|  | 3-hydroxybutyrate, mmol/L |  | 158 | -0.152 (0.073) | **0.04** | -0.089 (0.078) | 0.26 |
| **Fatty acids^c^** | |  |  |  |  |  |  |
|  | Total fatty acids, mmol/L |  | 130 | -0.217 (0.061) | **<0.001** | -0.159 (0.069) | **0.02** |
|  | Omega-3 fatty acids, mmol/L |  | 130 | -0.173 (0.067) | **0.01** | -0.154 (0.074) | **0.04** |
|  | Docosahexaenoic acid (DHA), mmol/L |  | 130 | -0.057 (0.060) | 0.35 |  |  |
|  | Omega-6 fatty acids, mmol/L |  | 130 | -0.322 (0.062) | **<0.001** | -0.288 (0.070) | **<0.001** |
|  | Linoleic acid, mmol/L |  | 130 | -0.262 (0.061) | **<0.001** | -0.232 (0.068) | **0.001** |
|  | Polyunsaturated fatty acids, mmol/L |  | 130 | -0.319 (0.059) | **<0.001** | -0.283 (0.067) | **<0.001** |
|  | Monounsaturated fatty acids, mmol/L |  | 130 | -0.211 (0.057) | **<0.001** | -0.114 (0.065) | 0.08 |
|  | Saturated fatty acids, mmol/L |  | 130 | -0.103 (0.072) | 0.15 |  |  |
|  | Fatty acid chain length |  | 130 | 0.414 (0.099) | **<0.001** | 0.437 (0.104) | **<0.001** |
| **Fatty acid ratios, relative to total fatty acids^c^** | |  |  |  |  |  |  |
|  | Omega-3 fatty acids, % |  | 130 | -0.017 (0.085) | 0.84 |  |  |
|  | Docosahexaenoic acid (DHA), % |  | 130 | 0.093 (0.073) | 0.20 |  |  |
|  | Omega-6 fatty acids, % |  | 130 | -0.170 (0.073) | **0.02** | -0.206 (0.079) | **0.01** |
|  | Linoleic acid, % |  | 130 | -0.088 (0.060) | 0.14 |  |  |
|  | Polyunsaturated fatty acids, % |  | 130 | -0.166 (0.073) | **0.02** | -0.207 (0.080) | **0.01** |
|  | Monounsaturated fatty acids, % |  | 130 | -0.131 (0.060) | **0.03** | -0.027 (0.066) | 0.68 |
|  | Saturated fatty acids, % |  | 130 | 0.378 (0.096) | **<0.001** | 0.305 (0.100) | **0.002** |
|  | Unsaturation degree, double bonds per fatty acids |  | 130 | -0.125 (0.078) | 0.11 | -0.137 (0.085) | 0.11 |
| **Fluid balance** | |  |  |  |  |  |  |
|  | Creatinine, mmol/L |  | 162 | -0.090 (0.050) | 0.07 |  |  |
|  |  | Men | 81 | -0.033 (0.069) | 0.63 |  |  |
|  |  | Women | 81 | -0.146 (0.072) | **0.04** | -0.110 (0.078) | 0.16 |
|  | Albumin, signal area |  | 162 | -0.069 (0.076) | 0.37 |  |  |
| **Inflammation** | |  |  |  |  |  |  |
|  | Glycoprotein acetyls, mainly a1-acid glycoprotein, mmol/L |  | 162 | -0.155 (0.058) | **0.008** |  |  |
|  |  | Men | 81 | -0.221 (0.082) | **0.007** | -0.126 (0.092) | 0.16 |
|  |  | Women | 81 | -0.089 (0.083) | 0.28 |  |  |
| **Apolipoproteins^c^** | |  |  |  |  |  |  |
|  | Apolipoprotein B, g/L |  | 134 | -0.311 (0.048) | **<0.001** | -0.225 (0.056) | **<0.001** |
|  | Apolipoprotein A1, g/L |  | 134 | -0.147 (0.052) | **0.004** | -0.210 (0.059) | **<0.001** |
|  |  | Men | 65 | -0.009 (0.071) | 0.90 |  |  |
|  |  | Women | 69 | -0.278 (0.072) | **<0.001** | -0.308 (0.081) | **<0.001** |
| **Lipids^c^** | |  |  |  |  |  |  |
|  | Triglycerides, mmol/L |  | 134 | -0.179 (0.058) | **0.002** |  |  |
|  |  | Men | 65 | -0.290 (0.086) | **0.001** | -0.200 (0.097) | **0.04** |
|  |  | Women | 69 | -0.075 (0.075) | 0.32 |  |  |
|  | Phosphoglycerides, mmol/L |  | 130 | -0.258 (0.068) | **<0.001** | -0.276 (0.075) | **<0.001** |
|  |  | Men | 63 | -0.246 (0.094) | **0.009** | -0.323 (0.107) | **0.003** |
|  |  | Women | 67 | -0.269 (0.097) | **0.006** | -0.248 (0.105) | **0.02** |
|  | Cholines, mmol/L |  | 130 | -0.189 (0.064) | **0.003** | -0.218 (0.071) | **0.002** |
|  |  | Men | 63 | -0.171 (0.090) | 0.06 |  |  |
|  |  | Women | 67 | -0.205 (0.091) | **0.02** | -0.183 (0.098) | 0.06 |
|  | Sphingomyelins, mmol/L |  | 130 | -0.258 (0.051) | **<0.001** | -0.286 (0.059) | **<0.001** |
|  |  | Men | 63 | -0.182 (0.067) | **0.006** | -0.281 (0.083) | **0.001** |
|  |  | Women | 67 | -0.329 (0.076) | **<0.001** | -0.313 (0.083) | **<0.001** |
| **Total lipid concentrations in lipoprotein subclasses^c^** | |  |  |  |  |  |  |
|  | Extremely large VLDL, mmol/L |  | 115 | -0.244 (0.079) | **0.002** | -0.235 (0.085) | **0.006** |
|  | Very large VLDL, mmol/L |  | 103 | -0.306 (0.084) | **<0.001** | -0.277 (0.091) | **0.002** |
|  | Large VLDL, mmol/L |  | 119 | -0.288 (0.066) | **<0.001** | -0.195 (0.072) | **0.007** |
|  | Medium VLDL, mmol/L |  | 134 | -0.232 (0.056) | **<0.001** | -0.114 (0.063) | 0.07 |
|  | Small VLDL, mmol/L |  | 134 | -0.284 (0.055) | **<0.001** | -0.176 (0.062) | **0.005** |
|  | Very small VLDL, mmol/L |  | 134 | -0.283 (0.059) | **<0.001** | -0.239 (0.067) | **<0.001** |
|  | IDL, mmol/L |  | 134 | -0.245 (0.051) | **<0.001** | -0.222 (0.059) | **<0.001** |
|  | Large LDL, mmol/L |  | 134 | -0.232 (0.052) | **<0.001** | -0.188 (0.060) | **0.002** |
|  | Medium LDL, mmol/L |  | 134 | -0.240 (0.055) | **<0.001** | -0.179 (0.063) | **0.004** |
|  | Small LDL, mmol/L |  | 134 | -0.266 (0.054) | **<0.001** | -0.205 (0.061) | **0.001** |
|  | Very large HDL, mmol/L |  | 133 | 0.069 (0.061) | 0.26 |  |  |
|  |  | Men | 64 | 0.217 (0.094) | **0.02** | 0.025 (0.096) | 0.80 |
|  |  | Women | 69 | -0.068 (0.075) | 0.36 |  |  |
|  | Large HDL, mmol/L |  | 126 | 0.091 (0.046) | 0.05 |  |  |
|  |  | Men | 59 | 0.241 (0.079) | **0.002** | 0.077 (0.087) | 0.38 |
|  |  | Women | 67 | -0.042 (0.048) | 0.38 |  |  |
|  | Medium HDL, mmol/L |  | 134 | -0.115 (0.068) | 0.09 |  |  |
|  |  | Men | 65 | 0.025 (0.091) | 0.79 |  |  |
|  |  | Women | 69 | -0.247 (0.098) | **0.01** | -0.246 (0.105) | **0.02** |
|  | Small HDL, mmol/L |  | 134 | -0.200 (0.088) | **0.02** | -0.111 (0.092) | 0.228 |
|  |  | Men | 65 | -0.215 (0.120) | 0.07 |  |  |
|  |  | Women | 69 | -0.185 (0.129) | 0.15 |  |  |
| **Lipoprotein particle size^c^** | |  |  |  |  |  |  |
|  | VLDL diameter, nm |  | 134 | -0.136 (0.067) | **0.04** | -0.032 (0.073) | 0.66 |
|  | LDL diameter, nm |  | 134 | 0.115 (0.095) | 0.23 |  |  |
|  | HDL diameter, nm |  | 134 | 0.108 (0.050) | **0.03** | -0.051 (0.054) | 0.35 |
|  |  | Men | 65 | 0.258 (0.077) | **0.001** | 0.081 (0.081) | 0.32 |
|  |  | Women | 69 | -0.032 (0.060) | 0.59 |  |  |
| **Cholesterol^c^** | |  |  |  |  |  |  |
|  | Total cholesterol, mmol/L |  | 134 | -0.296 (0.051) | **<0.001** | -0.279 (0.059) | **<0.001** |
|  | VLDL cholesterol, mmol/L |  | 134 | -0.301 (0.059) | **<0.001** | -0.223 (0.067) | **0.001** |
|  | IDL cholesterol, mmol/L |  | 134 | -0.265 (0.051) | **<0.001** | -0.249 (0.059) | **<0.001** |
|  | LDL cholesterol, mmol/L |  | 134 | -0.246 (0.054) | **<0.001** | -0.197 (0.061) | **0.001** |
|  | HDL cholesterol, mmol/L |  | 134 | 0.005 (0.043) | 0.90 |  |  |
|  |  | Men | 65 | 0.140 (0.062) | **0.02** | 0.015 (0.072) | 0.83 |
|  |  | Women | 69 | -0.122 (0.055) | **0.03** | -0.174 (0.066) | **0.008** |
|  | HDL2 cholesterol, mmol/L |  | 134 | 0.023 (0.042) | 0.58 |  |  |
|  |  | Men | 65 | 0.163 (0.061) | **0.008** | 0.036 (0.073) | 0.62 |
|  |  | Women | 69 | -0.108 (0.055) | **0.05** | -0.160 (0.066) | **0.02** |
|  | HDL3 cholesterol, mmol/L |  | 134 | -0.023 (0.074) | 0.75 |  |  |
|  |  | Men | 65 | -0.005 (0.104) | 0.96 |  |  |
|  |  | Women | 69 | -0.041 (0.107) | 0.70 |  |  |

^a^ P value refers to difference between baseline and end. ^b^ P value refers to difference between baseline and end after adjustment for weight loss. ^c^ Individuals using lipid-lowering agents were removed before analysis. All metabolite concentrations were natural log-transformed and scaled to standard deviation units before analysis. Parameters were analysed separately in men and women if there was a significant gender-difference at baseline. VLDL, very low density lipoprotein; IDL, intermediate density lipoprotein; LDL, low density lipoprotein; HDL, high density lipoprotein.

**Suplementary Figures**

**Supplementary Figure 1.** Effect of the intervention on insulin levels.

**Supplementary Figure 2.** Association of ^1^H-NMR metabolites with PC’s identified using PCA analysis. The colour of the cells represents the magnitude of the effect.
